# Supplementary material for: Exclusion Diets in Functional Dyspepsia
Source: Nutrients. 2022 May 14;14(10):2057. doi: 10.3390/nu14102057 (PMC9144481; doi:10.3390/nu14102057)
Supplement: Supplementary file 1 [file nutrients-14-02057-s001.zip › nutrients-1728180-supplementary.pdf]

Table S1. NHLBI Quality Assessment Tool for Controlled Intervention Studies

Criteria	Shahbazkhani et al. (2020)	Elli et al. (2016)	Potter et al. (2020)	Goyal et al. (2021)
1. Was the study described as randomized, a randomized trial, a randomized clinical trial, or an RCT?	Yes	Yes	Yes	Yes
2. Was the method of randomization adequate (i.e., use of randomly generated assignment)?	Yes	Yes	Yes	Yes
3. Was the treatment allocation concealed (so that assignments could not be predicted)?	Yes	Yes	Yes	Yes
4. Were study participants and providers blinded to treatment group assignment?	No	No	No	No
5. Were the people assessing the outcomes blinded to the participants' group assignments?	Yes	Yes	Yes	Yes
6. Were the groups similar at baseline on important characteristics that could affect outcomes (e.g., demographics, risk factors, co-morbid conditions)?	Yes	NR	NR	Yes
7. Was the overall drop-out rate from the study at endpoint 20% or lower of the number allocated to treatment?	No	Yes	Yes	No
8. Was the differential drop-out rate (between treatment groups) at endpoint 15 percentage points or lower?	Yes	Yes	Yes	Yes
9. Was there high adherence to the intervention protocols for each treatment group?	NR	NR	NR	Yes
10. Were other interventions avoided or similar in the groups (e.g., similar background treatments)?	Yes	NR	NR	NR
11. Were outcomes assessed using valid and reliable measures, implemented consistently across all study participants?	Yes	Yes	Yes	Yes
12. Did the authors report that the sample size was sufficiently large to be able to detect a difference in the main outcome between groups with at least 80% power?	NR	Yes	No	NR
13. Were outcomes reported or subgroups analyzed prespecified (i.e., identified before analyses were conducted)?	No	No	No	Yes
14. Were all randomized participants analyzed in the group to which they were originally assigned, i.e., did they use an intention-to-treat analysis?	Yes	Yes	Yes	Yes
Rating	Good	Good	Fair	Good

NR: Not Reported

Table S2. The Newcastle-Ottawa Scale (NOS) for assessing the quality of case-control studies

Study	Selection				Comparability	Exposure			Score	
	**Adequate definition**	**Representativeness of the cases**	**Selection of Controls**	**Definition of controls**	**Comparativeness of the cases and controls on the basis of the design or analysis**	**Ascertainment of exposure**	**Same method of ascertainment for cases and controls**	**Non-Response rate**		
Du et al. (2017)	*	*	*	*	⋆	*	*	-	(7)	Good
Staudacher et al. (2021)	*	*	*	*	**	*	*	-	(8)	Good

Table S3. NHLBI Quality Assessment Tool for Before-After Studies with no control group

**Criteria**	Tejedor et al. (2020)
1. Was the study question or objective clearly stated?	Yes
2. Were eligibility/ selection criteria for the study population prespecified and clearly described?	Yes
3. Were the participants in the study representative of those who would be eligible for the test/service/intervention in the general or clinical population of interest?	Yes
4. Were all eligible participants that met the prespecified entry criteria enrolled?	No
5. Was the sample size sufficiently large to provide confidence findings?	Yes
6. Was the test/service/intervention clearly described and delivered consistently across the study population?	Yes
7. Were the outcome measures prespecified, clearly defined, valid, reliable, and assessed consistently across all study participants?	CD
8. Were the people assessing the outcomes blinded to the participants' exposures/interventions?	CD
9. Was the loss to follow-up after baseline 20% or less? Were those lost to follow-up accounted for in the analysis?	CD
10. Did the statistical methods examine changes in outcome measures from before to after the intervention? Were statistical tests done that provided p values for the pre-to-post changes?	Yes
11. Were outcome measures of interest taken multiple times before the intervention and multiple times after the intervention (i.e., did they use an interrupted time-series design)?	CD
12. If the intervention was conducted at a group level (e.g., a whole hospital, a community, etc.) did the statistical analysis take into account the use of individual-level data to determine effects at the group level?	Yes
Rating	Fair

CD: can't determine

Table S4. The Newcastle-Ottawa Scale (NOS) for assessing the quality of cross-sectional studies

Study	Selection				Comparability	Outcome		Score	Rating
	Sample representativeness	Sample size	Non-Respondents	Ascertainment of the exposure (risk factor)	Comparability	Assessment of the outcome	Statistical test		
Adibi et al. (2021)	*	*	*	**	*	*	*	(9)	Good
